# The effect of non traditional teaching methods in entrepreneurship education on students entrepreneurial interest and business startups: A data article

**DOI:** 10.1016/j.dib.2018.04.142

**Published:** 2018-05-08

**Authors:** Maxwell Olokundun, Chinonye Love Moses, Oluwole Iyiola, Stephen Ibidunni, Mercy Ogbari, Fred Peter, Taiye Borishade

**Affiliations:** Covenant University, Nigeria

**Keywords:** Entrepreneurship education, Experiential teaching methods, Entrepreneurial interest, Student business startups

## Abstract

Traditional methods of teaching entrepreneurship in universities involves more theoretical approaches which are less effective in motivating considerations for an entrepreneurship career. This owes to the fact that such techniques essentially make students develop a dormant attitude rather than active participation. Expert views suggest that experiential entrepreneurship teaching methods in universities which involve practical activities and active participation can be considered salient to students’ development of entrepreneurial interest an business startup potentials. This present study presents data on the extent to which experiential teaching methods in entrepreneurship adopted by Nigerian universities stimulate students’ entrepreneurial interest and business startups. Data have been gathered following a descriptive cross-sectional quantitative survey conducted among university students (*N* = 600) of four selected institutions in Nigeria offering a degree programme in entrepreneurship. Hierarchical Multiple Regression Analysis was used in confirming the hypothesis proposed in the study using the Statistical Package for Social Sciences (SPSS) version 22.The findings from the analysis showed that the adoption of experiential practical activities considered as best practices in entrepreneurship teaching in Nigerian universities can stimulate students’ interest and drive for engaging in business start-up activities even as undergraduates. The field data set is made extensively available to allow for critical investigation.

**Specification Table**

**Table:** 

**Subject area**	Business, Management
**More specific subject area**	Business and Entrepreneurship education
**Type of data**	Table
**How data was acquired**	Researcher-made questionnaire analysis
**Data format**	Raw, analyzed, Inferential statistical data
**Experimental factors**	Sample consisted of university students in Nigeria. The researcher-made questionnaire which contained data on NonTraditional Teaching Methods in Entrepreneurship Education and Students’ Entrepreneurial Interest and Business Startups were completed
**Experimental features**	Traditional approach to teaching entrepreneurship in the university context is a major constraint of entrepreneurial development of university students.
**Data source location**	South west Nigeria
**Data accessibility**	Data is included in this article

**Value of data**

• The data presented revealed that experiential methods in entrepreneurship stimulate students’ entrepreneurial interest and propensity for business start-up.
• The data also showed that experiential approaches are suitable in teaching entrepreneurship to university students.
• The results of this study can be used to improve teaching and learning practices in university entrepreneurship education.

## 1 Data

The data for this research was collected from university students in four selected Nigerian institutions offering a degree programme in entrepreneurship. A total of six hundred (600) copies of questionnaire were distributed and five hundred and sixty four (564) copies were returned representing ninety four percent (94%) response rate. The study adopted descriptive cross sectional survey research design in which the research questionnaire was administered to respondents based on purposive, stratified and simple random sampling techniques. Table 1 below shows the allocation of copies of the questionnaire based on proportionate ratio.

**Table 1:** Allocation of copies of questionnaire.

School name	Population	Proportionate ratio	Copies of questionnaire
Federal University Of Agriculture Abeokuta	15,500	15,500 ÷ 50,900 × 600 = 183	183
Federal University Of Technology Akure	25,400	25,400 ÷ 50,900 × 600 = 288	288
Lead City University Ibadan	4300	4300 ÷ 50,900 × 600 = 50	50
Joseph Ayo Babalola University	5700	5700 ÷ 50,900 × 600 = 79	79
Total	50,900		600

## 2 Experimental design, materials and methods

The focus of this study was to assess the degree to which exposure to nontraditional teaching methods impacts on students’ entrepreneurial interest and expression of entrepreneurial intentions in the Nigerian university context. Therefore, the variables employed in this study were entrepreneurship education, learning orientation and entrepreneurial intentions. Three items each were developed based on [1], [2], [3], [4] to measure these variables. Copies of questionnaire were distributed to collect quantitative data on the relationship between nontraditional teaching methods, entrepreneurial interest and business startups. Five Likert-scale questions ranging from strongly agree to strongly disagree was adopted (strongly agree = 5, agree = 4, undecided = 3, disagree = 2, strongly disagree = 1). Hierarchical Multiple Regression Analysis was used in confirming the hypothesis proposed in the study using the Statistical Package for Social Sciences (SPSS) version 22. The validity and reliability of the research instruments were analyzed using content validity and Cronbach Alpha Reliability Procedure. To ensure content validity experts on the subject matter of this study were provided with access to the measurement tool in order to provide feedback on the effectiveness of each question in measuring the constructs. Informed decisions were made based on their feedbacks. The test to determine the internal consistency of the research instrument was conducted on the retrieved copies of questionnaire with the aid of the Cronbach Alpha Reliability procedure (Table 2, Table 3).

**Table 2:** Reliability statistics.

Cronbach's alpha	N of items
856	40

**Table 3:** Model summary.

Model	R	R square	Adjusted R square	Standard error of the estimate	Change statistics				
					R square change	F change	df1	df2	Significant F change
1	.435^a^	.189	.188	.52972	.189	131.580	1	563	.000
2	.623^b^	.388	.385	.46088	.198	181.753	1	562	.000

a Predictors: (Constant), Experiential teaching methods.

b Predictors: (Constant), Experiential teaching methods, interest.

The result indicated that the instrument had a good internal consistency based on the Cronbach Alpha Coefficient value reported at .856.

### 2.1 Hypothesis testing

**H**_**01**_ Experiential teaching Methods in entrepreneurship do not stimulate students’ entrepreneurial interest and business start-up.

The test of hypothesis was to examine the effects of experiential teaching methods in entrepreneurship and students’ entrepreneurial interest for business startups. In the first step, the effect of experiential teaching methods in entrepreneurship on students’ business startups was assessed. The R-Square value is the degree of variation of the dependent variable, which can be predicted by the independent variable. Consequently, the analysis revealed that experiential teaching methods in entrepreneurship explained 18.8% variance in students’ business startups (*R*^2^ = .188, *F* (2, 563) = 131.580, *p* ˂ 0.05). In the second step, the mediating role of entrepreneurial interest was examined. The analysis showed that entrepreneurial interest was able to predict 38.5% variance in students’ business startups over and beyond the effects of experiential teaching methods in entrepreneurship (*R*^2^ = .385, *F* (1, 562) = 181.753, *p* < .05). The significance of the F-change was assessed and it was significant (0.000).

Table 4 above shows the results of the two models. The first model showed the effect of experiential teaching methods in entrepreneurship on students’ business startups. The F-value is calculated as the Mean Square Regression (36.923) divided by the Mean Square Residual (.281), yielding F = 131.580. From this results, model 1 in the table is statistically significant (Sig = 0.000). The second model examined the effect of experiential teaching methods in entrepreneurship and students’ entrepreneurial interest to engage in business startups. The F-value is calculated as the Mean Square Regression (37.765) divided by the Mean Square Residual (.212), yielding F = 177.789 at an acceptable significant level of .000.

**Table 4:** Analysis of variance (experiential teaching methods student's interest and business startup).

Model		Sum of squares	Degree of freedom	Mean square	F	Significance
1	Regression	36.923	1	36.923	131.580	.000^a^
	Residual	157.983	563	.281		
	Total	194.905	564			
2	Regression	75.529	2	37.765	177.789	.000^b^
	Residual	119.376	562	.212		
	Total	194.905	564			

^c^ Dependent Variable: business start-up.

a Predictors: (constant), teaching methods.

b Predictors: (constant), teaching methods, interest.

Table 5 below shows the contributions of the independent and mediating variables to the variance in the dependent variable and their levels of significance.

**Table 5:** Coefficients^a^ (teaching methods and student's interest).

Model		Unstandardized coefficients		Standardized coefficients	T	Sig.	Correlations			Collinearity statistics	
		B	Std. error	Beta			Zero-order	Partial	Part	Tolerance	VIF
1	(Constant)	2.323	.146		15.920	.000					
	Teaching methods	.416	.036	.435	11.471	.000	.435	.435	.435	1.000	1.000
2	(Constant)	.846	.168		5.043	.000					
	Teaching methods	.213	.035	.223	6.083	.000	.435	.249	.201	.814	1.228
	Interest	.580	.043	.493	13.482	.000	.589	.494	.445	.814	1.228

a Dependent variable: business- start up.

Based on the results in model 2, the table above revealed the contributions of experiential teaching methods in entrepreneurship on students’ entrepreneurial interest and business start-ups and the levels of significance. (Non-traditional teaching methods; β = .213; t = 6.083; p < .001, interest; β = .580; t = 13.482; p < .05).

### 2.2 Conclusion and implications of the study

The data presented was based on a study to examine the effect of experiential teaching methods and university entrepreneurial interest on students’ business startup. It is important to note that experiential teaching methods are getting much attention from educators and universities as regards entrepreneurship teaching. The increasing necessity for university students to develop entrepreneurial capabilities while in school compels universities to realize the essence of experiential teaching methods and its effect on students ‘entrepreneurial interest and business startups. This has far reaching implications for both the universities, entrepreneurship educators and undergraduate students in Nigeria. Regardless of the peculiar institutional approach to entrepreneurship teaching in various Nigerian universities, experiential teaching methods should be adopted as an institutional culture. Therefore, the data described in this article is made widely accessible to facilitate critical or extended analysis.

### 2.3 Ethical considerations

The researchers established that the respondents were sufficiently informed about the goal of this research and they were well-informed about the process and participation in the research. Respondents were given the chance to stay anonymous and their responses were treated privately. Consent was obtained from the proper authorities in the organisations preceding the distribution of the copies of questionnaire.
