# Supplementary material for: The effect of non traditional teaching methods in entrepreneurship education on students entrepreneurial interest and business startups: A data article
Source: Data Brief. 2018 May 8;19:16–20. doi: 10.1016/j.dib.2018.04.142 (PMC5992968; doi:10.1016/j.dib.2018.04.142)
Supplement: Supplementary file 1 — Supplementary material [file mmc1.docx]

**Examining the Effect of Non Traditional Teaching Methods in Entrepreneurship Education on Students’ Entrepreneurial Interest and Business Startups**

Maxwell Olokundun, Chinonye, Love Moses , Oluwole Iyiola , Mercy Ejovwokeoghene Ogbari, Stephen Ayodotun Ibidunni and Fred Peter

**Abstract**

Traditional methods of teaching entrepreneurship in universities involves more theoretical approaches which are less effective in motivating considerations for an entrepreneurship career. This owes to the fact that such techniques essentially make students develop a dormant attitude rather than active participation. Expert views suggest that experiential entrepreneurship teaching methods in universities which involve practical activities and active participation can be considered salient to students’ development of entrepreneurial interest an business startup potentials. This present study seeks to assess the extent to which experiential teaching methods in entrepreneurship adopted by Nigerian universities stimulate students’ entrepreneurial interest and business startups. Data have been gathered following a descriptive cross-sectional quantitative survey conducted among university students (N=600) of four selected institutions in Nigeria offering a degree programme in entrepreneurship. Hierarchical Multiple Regression Analysis was used in confirming the hypothesis proposed in the study using the Statistical Package for Social Sciences (SPSS) version 22.The findings from the analysis showed that the adoption of experiential practical activities considered as best practices in entrepreneurship teaching in Nigerian universities can stimulate students’ interest and drive for engaging in business start-up activities even as undergraduates. It was recommended that experiential teaching methods such as invitation of guest speakers, individual and group project, business simulations activities, entrepreneurship related talks by real life mentors, entrepreneurial excursions and other more exciting and interesting practices in entrepreneurship teaching should be adopted by Nigerian universities in order to stimulate students’ entrepreneurial interest for business start-ups.

**Keywords**: Entrepreneurship education, Experiential Teaching Methods, Entrepreneurial Interest, Student Business Startups

**Introduction**

A major aim of entrepreneurship education in the university context is to make individuals ready to perform as entrepreneurs; hence experiential methods in entrepreneurship education enable tryouts by practicing entrepreneurship in an organized environment (Ahmad *et al*. 2004; Moses, Olokundun, Akinbode & Agboola*.* 2016) Kirby (2004) opined that though most entrepreneurship instructors associate their courses with business startup, but quite conversely what they do in the real sense is to teach about entrepreneurship without practical activities that motivate business creation. This suggests that traditional methods of teaching entrepreneurship, which involves more theoretical approaches, are less effective in motivating considerations for an entrepreneurship career (Agbonlahor, 2016). This owes to the fact that such techniques essentially make students develop a dormant attitude rather than active participation (Mwasalwiba, 2010 ; Odukoya *et al* 2017). Therefore, it is possible that experiential entrepreneurship teaching methods which involve practical activities and active participation can be considered salient to university students’ business startup potentials (Janes *et al*. 2016). However, entrepreneurial interest can influence an individual's ability to acquire knowledge and sense of values. Hence, engagement of experiential teaching methods regardless, entrepreneurial interest may be required to motivate students’ business startup propensity (Olokundun *et al*. 2017) Consequently, studies such as Arasti *et al* (2012), Rae and Carswell (2001), and Shepherd and Douglas (1997) have studied non-traditional teaching methods in entrepreneurship and their appropriateness for entrepreneurial needs of graduate students. However, there is a paucity of research on experiential teaching methods in entrepreneurship education and the effects on students’ entrepreneurial interest and business startups particularly in Nigerian Universities. Therefore the main objective of this study is to assess the extent to which experiential teaching methods in entrepreneurship adopted by Nigerian universities stimulate students’ interest in engaging in entrepreneurial activities with emphasis on business startups particularly during the course of an entrepreneurship programme.

**1.0 Literature Review and Hypothesis**

The concept of entrepreneurship has become a phenomenon. Entrepreneurship has emerged as the most potent economic force the world has ever experienced (Kuratko, 2005; Fairlie & Fossen, 2017). Drucker (1985) supported by Bwemelo (2017) defined entrepreneurship as a perceptiveness to change and the entrepreneur as one, who always searches for change, responds to and exploits it as an opportunity. Drucker notes that entrepreneurship is practice behaviour. Aina and Salako (2008) described entrepreneurship as the willingness and ability of an individual to seek out investment opportunities and take advantage of scarce resources to exploit the opportunities profitably. It is the process of creating something new with value by devoting the necessary time and efforts, assuming the accompanying financial social risks and receiving the resulting reward. This was endorsed by the study of Lea, Kumar and Matthias (2017) who opined that entrepreneurship is actually concerned with the identification of gaps and business opportunities in one’s immediate environment and bringing together the necessary resources in an innovative way to fill these gaps and in the process gain personal rewards (which may or may not be for profit motives). In the same vein Römer-Paakkanen and Suonpää (2017) stated that entrepreneurship is the willingness and the ability of an individual to seek out a new investment opportunity, establish an enterprise and successfully run it either for profit making or social benefit. It is important to note that entrepreneurship as a dynamic process of vision, change and creation, requires to be taught for the transfer of its skills and knowledge from an expert to someone else (Kuratko, 2005). Therefore, the concept of entrepreneurship education plays an important role in the entrepreneurial development of individuals.

The teaching methods engaged in entrepreneurship education should stimulate students’ interest in entrepreneurship and activities involving business startups (Ruškytė1 & Navickas 2017). This is in line with the study of Arasti, falavarjani, and Imanipour (2012) that focused on the suitable teaching methods in entrepreneurship education by carrying out two qualitative studies. The authors concluded that the appropriate teaching methods that stimulate students’ entrepreneurial interest are group project, case study, individual project, development of a new business startup project and problem-solving. In the same vein, Malach and Malach (2014) examined the experiential entrepre­neurship education approach highlighting the “Start Your Own Business” Assignment in the context of the entrepreneurship course offered to over 200 undergraduate students per year at the Haskayne School of Business, University of Calgary, Canada. The findings of the study suggests that experiential education in entrepreneurship courses, stimulate students’ interest and convey both substantive, theoretical knowledge and intan­gible learning experiences best absorbed through active partici­pation. The authors concluded that starting and operating a business is a unique, educational experience which allows students to apply the substantive knowl­edge gained in entrepreneurship courses to a real business. Similarly, Canziani, Welsh, Hsieh, and Tuller (2015) investigated the effectiveness of different teaching methods in entrepreneurship. The research focused on three learning design choices namely; experiential learning, use of teamwork and focus on quantitative methods. The paper examined teaching methods that could contribute to raising student scores on constructs of change, risk taking, goal setting, feedback and achievement as measured by a customized entrepreneurial propensity survey. The researchers asserted that experiential and practical oriented teaching methods stimulate student interest for entrepreneurial venturing.

Penanluna, Peneluna, and Jones (2012) examined the contextual contrasts in the development and delivery of enterprise education in higher education globally. Utilizing data gotten from an online survey conducted on enterprise educators, the authors concluded that there are low levels of business start-up activities among students during enterprise education and one year after graduation. This trend maybe as a result of loss of entrepreneurial interest by students at graduation which endorses the study of Rodriguez, Chen, Sheppard, Leifer, and Jin (2015) who explored the reasons for some engineering graduates who co-founded or started a company may no longer have an entrepreneurial interest. The participants in this study were 484 alumni who received their undergraduate engineering degrees in 2007 from four different universities in the United States of America. The authors argued that one of the factors responsible for loss of entrepreneurial interest is that despite exposure to entrepreneurship education, graduates appear to choose positions that would support career advancement. This may be consequent on the use of traditional methods connoting absence of practical activities in entrepreneurship teaching. Based on the above discussions, the research model below has been sketched. The model illustrates projected relationships between variables of the hypothesis discussed in the following section.

**H1**

Student Business Startup

Students’ Entrepreneurial Interest

Y

Experiential

Teaching Methods

**Figure 1: Research framework**

**1.1 Experiential Teaching Methods, Entrepreneurial Interest and Business Start-ups**

As noted by Hidi *et al*. (1992) and Osakede, *et al* (2017) it would be quite an extraordinary and challenging task for educators, to take cognizance of the learners’ entrepreneurial interest given the time limitations and the class sizes instructors have to work with. However, experiential teaching methods therefore, create the environment that stimulates entrepreneurial interest for the course (Wang & Wong, 2004). The aforementioned calls for closer examination in university entrepreneurship education because; creating an environment that leverages upon the adoption of experiential methods in entrepreneurship teaching, could trigger entrepreneurial interest among students (Mitchell, 1993; Mohd Zaki, *et al* 2017). In line with Xiang *et al*. (2005) entrepreneurial interest that emerges out of the creation of important learning experiences in entrepreneurship teaching has the ability to motivate venture creation by individuals. Consequently, with specific reference to Nigerian universities, there is a probability that experiential teaching methods has profound contribution to make university students develop entrepreneurial interest and engage in business startup activities. On the basis of the above evidences, the following hypothesis has been developed.

*H1: Experiential teaching Methods in entrepreneurship do not stimulate students’ entrepreneurial interest and business startups.*

**2.0 Methodology**

**2.1 Sample and Data Collection**

The data for this research was collected from university students in four selected Nigerian institutions offering a degree programme in entrepreneurship. A total of six hundred (600) copies of questionnaire were distributed and five hundred and sixty four (564) copies were returned representing ninety four percent (94%) response rate. The study adopted descriptive cross sectional survey research design in which the research questionnaire was administered to respondents based on purposive, stratified and simple random sampling techniques. Table 1 below shows the allocation of copies of the questionnaire based on proportionate ratio.

**Table 1: Allocation of Copies of Questionnaire**

| School Name | Population | Proportionate Ratio | Copies of Questionnaire |
| --- | --- | --- | --- |
| Federal University Of Agriculture Abeokuta | 15,500 | 15,500÷50,900× 600 = 183 | 183 |
| Federal University Of Technology Akure | 25,400 | 25,400÷50,900×600 = 288 | 288 |
| Lead City University Ibadan | 4,300 | 4,300÷50,900×600 = 50 | 50 |
| Joseph Ayo Babalola University | 5700 | 5,700÷50,900×600 = 79 | 79 |
| Total | 50, 900 |  | 600 |

**Source: Field Study (2016)**

**2.2 Measures and Instruments**

The focus of this study was to assess the degree to which exposure to nontraditional teaching methods impacts on students’ entrepreneurial interest and expression of entrepreneurial intentions in the Nigerian university context. Therefore, the variables employed in this study were entrepreneurship education, learning orientation and entrepreneurial intentions. Three items each were developed based on literature to measure these variables. Copies of questionnaire were distributed to collect quantitative data on the relationship between nontraditional teaching methods, entrepreneurial interest and business startups, Five Likert-scale questions ranging from strongly agree to strongly disagree was adopted (strongly agree=5, agree=4, undecided=3, disagree=2, strongly disagree-1). In developing the survey questionnaire instrument, questions were modified from existing literature that relate to the study.

**2.3 Data Analysis Techniques**

Hierarchical Multiple Regression Analysis was used in confirming the hypothesis proposed in the study using the Statistical Package for Social Sciences (SPSS) version 22. The validity and reliability of the research instruments were analyzed using content validity and Cronbach Alpha Reliability Procedure. To ensure content validity experts on the subject matter of this study were provided with access to the measurement tool in order to provide feedback on the effectiveness of each question in measuring the constructs (Ghauri & Gronhaug, 2002). Informed decisions were made based on their feedbacks. The test to determine the internal consistency of the research instrument was conducted on the retrieved copies of questionnaire with the aid of the Cronbach Alpha Reliability procedure (Creswell *et al*. 2003).

**2.4: Results**

**2.41: Reliability Analysis:**

**Table 2: Reliability Statistics**

| Cronbach’s Alpha | N of Items |
| --- | --- |
| .856 | 40 |

Source: Field work, (2016)

The result indicated that the instrument had a good internal consistency based on the Cronbach Alpha Coefficient value reported at 0.856.

**2.42: Results of Hypothesis Testing**

**H_01_** Experiential teaching Methods in entrepreneurship do not stimulate students’ entrepreneurial interest and business start-up.

**Table 2: Model Summary**

| Model | R | R Square | Adjusted R Square | Std. Error of the Estimate | Change Statistics | | | | |
| --- | --- | --- | --- | --- | --- | --- | --- | --- | --- |
|  |  |  |  |  | R Square Change | F Change | df1 | df2 | Sig. F Change |
| 1 | .435^a^ | .189 | .188 | .52972 | .189 | 131.580 | 1 | 563 | .000 |
| 2 | .623^b^ | .388 | .385 | .46088 | .198 | 181.753 | 1 | 562 | .000 |
| **a. Predictors: (Constant), Experiential teaching methods** | | | | | | | | | |
| **b. Predictors: (Constant), Experiential teaching methods, interest** | | | | | | | | | |

**Source: Field Survey Result (2016)**

The test of hypothesis was to examine the effects of experiential teaching methods in entrepreneurship and students’ entrepreneurial interest for business startups. In the first step, the effect of experiential teaching methods in entrepreneurship on students’ business startups was assessed. The R-Square value is the degree of variation of the dependent variable, which can be predicted by the independent variable. Consequently, the analysis revealed that experiential teaching methods in entrepreneurship explained 18.8% variance in students’ business startups (*R*^2^ = .188, *F* (2, 563) = 131.580, *p* ˂ 0.05). In the second step, the mediating role of entrepreneurial interest was examined. The analysis showed that entrepreneurial interest was able to predict 38.5% variance in students’ business startups over and beyond the effects of experiential teaching methods in entrepreneurship (*R*^2^ = .385, *F* (1, 562) = 181.753, *p* ˂ 0.05). The significance of the F-change was assessed and it was significant (0.000).

**Table 3: ANOVA^c^ (Experiential Teaching Methods Student’s Interest and Business Startup)**

| Model | | Sum of Squares | Df | Mean Square | F | Sig. |
| --- | --- | --- | --- | --- | --- | --- |
| 1 | Regression | 36.923 | 1 | 36.923 | 131.580 | .000^a^ |
|  | Residual | 157.983 | 563 | .281 |  |  |
|  | Total | 194.905 | 564 |  |  |  |
| 2 | Regression | 75.529 | 2 | 37.765 | 177.789 | .000^b^ |
|  | Residual | 119.376 | 562 | .212 |  |  |
|  | Total | 194.905 | 564 |  |  |  |
| **a. Predictors: (Constant), teaching methods** | | | | | | |
| **b. Predictors: (Constant), teaching methods, interest** | | | | | | |
| **c. Dependent Variable: business start-up** | | | | | | |

**Source: Field Survey Result (2016)**

Table 3 above shows the results of the two models. The first model showed the effect of experiential teaching methods in entrepreneurship on students’ business startups. The F-value is calculated as the Mean Square Regression (36.923) divided by the Mean Square Residual (0.281), yielding F=131.580. From this results, model 1 in the table is statistically significant (Sig =.000). The second model examined the effect of experiential teaching methods in entrepreneurship and students’ entrepreneurial interest to engage in business startups. The F-value is calculated as the Mean Square Regression (37.765) divided by the Mean Square Residual (0.212), yielding F=177.789 at an acceptable significant level of .000. Since the results of the Anova in table 4.6.3b show a significant level of 0.000, the alternate hypothesis which states that ‘experiential teaching methods in entrepreneurship stimulates students’ entrepreneurial interest for business startups’ is therefore accepted, while the null hypothesis which states that experiential teaching methods in entrepreneurship do not stimulate students’ entrepreneurial interest for business startups’ is rejected.

| Table 4 below shows the contributions of the independent and mediating variables to the variance in the dependent variable and their levels of significance.  **Table 4 : Coefficients^a^ (Teaching Methods and Student’s Interest)** | | | | | | | | | | | |
| --- | --- | --- | --- | --- | --- | --- | --- | --- | --- | --- | --- |
| Model | | Unstandardized Coefficients | | Standardized Coefficients | T | Sig. | Correlations | | | Collinearity Statistics | |
|  |  | B | Std. Error | Beta |  |  | Zero-order | Partial | Part | Tolerance | VIF |
| 1 | (Constant) | 2.323 | .146 |  | 15.920 | .000 |  |  |  |  |  |
|  | Teaching methods | .416 | .036 | .435 | 11.471 | .000 | .435 | .435 | .435 | 1.000 | 1.000 |
| 2 | (Constant) | .846 | .168 |  | 5.043 | .000 |  |  |  |  |  |
|  | Teaching methods | .213 | .035 | .223 | 6.083 | .000 | .435 | .249 | .201 | .814 | 1.228 |
|  | Interest | .580 | .043 | .493 | 13.482 | .000 | .589 | .494 | .445 | .814 | 1.228 |
| 1. Dependent Variable: business- start up   **Source: Field Survey Result (2016)** | | | | | | | | | | | |

Based on the results in model 2, the table above revealed the contributions of experiential teaching methods in entrepreneurship on students’ entrepreneurial interest and business start-ups and the levels of significance. (Non-traditional teaching methods; β = .213; t=6.083; p<0.001, interest; β = .580; t=13.482; p<0.05).

**Decision**

The significance levels of all the variables are less than 0.05 and the level of significance of F change is also less than 0.001 (.0001). Based on the results above, it is therefore justified that the alternate hypothesis should be accepted, while the null hypothesis should be rejected. It can therefore be concluded that experiential teaching methods in entrepreneurship stimulate students’ entrepreneurial interest and business start-ups.

**Discussion of Results**

Findings from the test of hypothesis revealed that experiential methods in entrepreneurship teaching such as simulations, invitation of guest speakers, debates, case studies, and other practical teaching methods, stimulate students’ entrepreneurial interest and business start-ups substantiating intentions for an entrepreneurial career. This shows that the adoption of effective practical activities considered as best practices in entrepreneurship teaching in Nigerian universities can stimulate students’ entrepreneurial interest and drive for engaging in business start-up activities even as undergraduates. This is in line with the work of Arasti, Falavarjani, and Imanipour (2012) which showed that the appropriate teaching methods for teaching entrepreneurship include; group project, case study, individual project, venture creation project, and problem-solving. These aforementioned teaching methods are pointers to experiential approaches which are considered suitable in teaching entrepreneurship to university students considering their interests and business startup propensity. This also extends the results of the study of Olokundun *et al* (2014) which indicated that the engagement of experiential teaching methods has implications for students’ business start-up. To extend the findings of these studies, this research has also shown that with the use of experiential teaching methods students’ entrepreneurial interest can be stimulated for business startups during the course of an entrepreneurship programme. Although the findings of some studies such as Hamidi, Wennberg, and Berglund (2008), suggests that participation in an entrepreneurship programme is averse to the development of students ‘interests and business startup potentials. However, this study counters this stance particularly because findings from this research suggests that engaging experiential teaching methods and best practices in entrepreneurship teaching, may motivate students interest for business startups in the course of the programme. Therefore, this study provides valid evidence to show that adoption of experiential teaching methods in entrepreneurship can stimulate students’ entrepreneurial interest for business start-ups in Nigerian universities.

**Conclusion and Recommendations**

The aim of this study was to examine the effect of experiential teaching methods and university entrepreneurial interest on students’ business startup. It is important to note that experiential teaching methods are getting much attention from educators and universities as regards entrepreneurship teaching. This study revealed that experiential teaching methods have significant and positive impact on students’ entrepreneurial interest and business startups. The increasing necessity for university students to develop entrepreneurial capabilities while in school compels universities to realize the essence of experiential teaching methods and its effect on students ‘entrepreneurial interest and business startups. The present study involves far-reaching implications for both the universities, entrepreneurship educators and undergraduate students in Nigeria. Regardless of the peculiar institutional approach to entrepreneurship teaching in various Nigerian universities, the present study suggests experiential teaching methods should be adopted as an institutional culture. Experiential teaching methods in entrepreneurship education such as invitation of guest speakers, individual and group project, business simulations activities, entrepreneurship related talks by real life mentors, entrepreneurial excursions and other more exciting and interesting practices in entrepreneurship teaching should be adopted by Nigerian universities in order to stimulate students’ entrepreneurial interest and business start-ups. Training the trainer programs should be organized to better educate entrepreneurship educators about the role and implementation of experiential teaching methods to increase knowledge and skills required to do their jobs. The present study also found that undergraduates can engage in business startup activities in the course of an entrepreneurship programme. Thus, according to the result of this research, universities should create an atmosphere where students can engage in business startup activities as a team with fair cooperation with their colleagues.

**Acknowledgement**

The authors wish to appreciate the management of Covenant University for offering full sponsorship for this research work.

**References**

Ahmad, S.h.F., Baharun, R. & Rahman, S.H.A. (2004). Interest in Entrepreneurship: an exploratory study on engineering and technical students in entrepreneurship education and choosing entrepreneurship as a career. In Project Report. Faculty of Management and Human Resource Development, Skudai, Johor. (Unpublished). Universiti Teknologi Malaysia Institutional Repository. [Online] Available: <http://eprints.utm.my/2668/>.

Aina, B.S & Salako, H.A. (2008). Determinants of foreign direct investment in Nigeria: an empirical investigation. *CBN Economic and Financial Review* **39(1)**

Arasti, Z., Falavarjani, M.K., & Imanipour, N. (2012). A Study of Teaching Methods in Entrepreneurship Education for Graduate Students. *Journal of Higher Education Studies*, **2(1)**, 2-10.

Creswell, J. W., Plano Clark, V. L., Gutmann, M. L., & Hanson, W. E. (2003). Advanced Mixed Methods Research Designs. In A. Tashakkori & C. Teddlie (Eds.), Handbook of Mixed Methods In Social And Behavioral Research Thousand Oaks, CA: Sage**.** 209– 240.

Drucker, P.F. (1985). Innovation and Entrepreneurship: Practice and Principles. Heinemann, London. ISBN 10: 0887306187, ISBN 13: 9780887306181. *Education and Training Journal*, **46(9)**, 416–423.

Fairlie, R.W & Fossen, F.M. (2017). Opportunity versus Necessity Entrepreneurship: Two Components of Business Creation. Stanford Institute for Economic Policy Research Stanford University Stanford, CA 94305 (650) 725-1874.

Ghauri, P., & Gronhaug, K. (2002). *Research methods in business studies.* Edinburgh, UK: Pearson Education Limited. ISBN*-*13: 978-0273712046.

Hamidi, D. Y., Wennberg, K., & Berglund, H. (2008). Creativity In Entrepreneurship Education. *Journal of Small Business and Enterprise Development,* **15(2)**, 304-320..

Hidi, S., Renninger, K. A., & Krapp, A. (1992). The Present State of Interest Research. In K. A. Renninger, S. Hidi, & A. Krapp (Eds.), The Role of Interest In Learning and Development Hillsdale, NJ: Lawrence Erlbaum Associates.

Janes, W.I, Silvey D, & Dubrowski, A. ( 2016) Are Educators Actually Coaches? The Implication of Teaching and Learning Via Simulation In Education In Healthcare Professions. *Cureus,* **8(8)**, 734. DOI 10.7759/Cureus.734.

Kirby, D. (2004). Entrepreneurship Education: Can Business Schools Meet The Challenge? *Education and Training*, 468**(9)**, 510-19. .

Kuratko, D.F. (2005), “The Emergence Of Entrepreneurship Education: Development, Trends, and Challenges”, *Entrepreneurship Theory And Practice* **29(5)**, 577–598.

Lea, Z., Kumar, K., & Matthias, P. (2017). Entrepreneurship education at Indian Industrial Training Institutes – A Case Study of the Prescribed, Adopted and Enacted Curriculum in and around Bangalore. *International Journal for Research in Vocational Education and Training* 4 (1), 69-94

Mitchell, J.R. & D.A. Shepherd (2010), “To Thine Own Self Be True: Images of Self, Images of Opportunity, And Entrepreneurial Action,” *Journal Of Business Venturing*, **25,(1)**,138- 154.C

Mohd Zaki, S., Tajuddin, N., Asmui, M. (2016). Students’ Interest in Entrepreneurship. ***Gading Journal for The Social Sciences*, 19(1)**, 1–5

Moses, C.L., Olokundun, M.A., Akinnbode, M., Agboola, G.M. (2016) [Organizational Culture and Creativity In Entrepreneurship Teaching In Nigerian Secondary Education](https://scholar.google.com/citations?view_op=view_citation&hl=en&user=vvx7YugAAAAJ&citation_for_view=vvx7YugAAAAJ:kNdYIx-mwKoC). *Research Journal of Applied Sciences,* 11**(1)**: 586-591

Mwasalwiba, E.S. (2010). Entrepreneurship Education: A Review of Its Objectives, Teaching Methods, and Impact Indicators. Education and Training, **52(1)**, 20–47.

Odukoya, J.A., Adekeye, O & [Okunlola, O.](https://www.scopus.com/authid/detail.uri?authorId=57195961225&amp;eid=2-s2.0-85030540583) (2017). Assessing the Effectiveness of Mobile Learning Devices in Tertiary Institutions: The Experience of Undergraduates in a Nigerian Private University. [*International Journal of Interactive Mobile Technologies*](https://www.scopus.com/sourceid/21100394784?origin=recordpage), 11(4), 160-169

Olokundun, M.A., Ibidunni, A.S Peter, F., Amaihian, A., Moses, C.L., & Iyiola, O.O (2017). Experiential Pedagogy and Shared Vision: A Focus on Identification of Business Opportunities By Nigerian University Students. *Journal of Entrepreneurship Education*, **20(2)**, 1–12.

Osakede, U.A., Lawanson, A.O., & Sobowale, D.A. (2017) Entrepreneurial Interest and Academic Performance in Nigeria: Evidence from Undergraduate Students in the University of Ibadan. *Journal of Innovation and Entrepreneurship,.* **6(19)**, 1–15

Rae, D. & Carswell, M. (2001). Towards a conceptual understanding of entrepreneurial learning, *Journal of Small Business and Enterprise Development*, **8(2)**, 150 – 158.

Römer-Paakkanen, T. & Suonpää, M (2017). Multiple Objectives and Means of Entrepreneurship Education at Finnish Universities of Applied Sciences. Haaga-Helia University of Applied Sciences. ISSN: 2342-2939 (pdf) ISBN: 978-952-7225-82-0 (pdf)

Ruškytė1, D. & Navickas, V. (2017). Efficiency of Teaching and Learning Methods for Development of Learner Entrepreneurship. *Pedagogy* **126 (2)**, 168–184

Shepherd, D.A., & Douglas, E.J. (1997), Is Management Education Developing or Killing the Entrepreneurial Spirit? Proceedings of the 1997 USASBE Annual National Conference Entrepreneurship: The Engine of Global Economic Development, San Francisco, California.Wang, C. K., & Wong, P. K. (2004).Entrepreneurial Interest of University Students In Singapoe. *Technovation*, **24(1)***,* 163–172.

Xiang, P., Chen, A., & Bruene, A. (2005). Interactive impact of intrinsic motivators and extrinsic rewards on behavior and motivation outcomes. *Journal of Teaching in Physical Education,* **24(1)***,* 179-197.
